# Preparation and Properties of Hollow Glass Microspheres/Dicyclopentadiene Phenol Epoxy Resin Composite Materials

**DOI:** 10.3390/ma16103768

**Published:** 2023-05-16

**Authors:** Jiadong Lu, Songli Zhang, Leizhi Zhang, Chenxi Wang, Chunying Min

**Affiliations:** 1 School of Materials Science & Engineering, Jiangsu University, Zhenjiang 212013, China; 2212005036@stmail.ujs.edu.cn (J.L.); 2222105109@stmail.ujs.edu.cn (C.W.); mj790206@126.com (C.M.); 2 School of Civil Engineering, Southeast University, Nanjing 211189, China; leizhizhang@seu.edu.cn

**Keywords:** electronic packaging materials, epoxy resin, dielectric properties, hollow glass microspheres

## Abstract

With the development of the integrated circuit and chip industry, electronic products and their components are becoming increasingly miniaturized, high-frequency, and low-loss. These demand higher requirements for the dielectric properties and other aspects of epoxy resins to develop a novel epoxy resin system that meets the needs of current development. This paper employs ethyl phenylacetate cured dicyclopentadiene phenol (DCPD) epoxy resin as the matrix and incorporates KH550 coupling-agent-treated SiO_2_ hollow glass microspheres to produce composite materials with low dielectric, high heat resistance, and high modulus. These materials are applied as insulation films for high density interconnect (HDI) and substrate-like printed circuit board (SLP) boards. The Fourier transform infrared spectroscopy (FTIR) technique was used to characterize the reaction between the coupling agent and HGM, as well as the curing reaction between the epoxy resin and ethyl phenylacetate. The curing process of the DCPD epoxy resin system was determined using differential scanning calorimetry (DSC). The various properties of the composite material with different HGM contents were tested, and the mechanism of the impact of HGM on the properties of the composite material was discussed. The results indicate that the prepared epoxy resin composite material exhibits good comprehensive performance when the HGM content is 10 wt.%. The dielectric constant at 10 MHz is 2.39, with a dielectric loss of 0.018. The thermal conductivity is 0.1872 Wm^−1^ k^−1^, the coefficient of thermal expansion is 64.31 ppm/K, the glass transition temperature is 172 °C, and the elastic modulus is 1221.13 MPa.

## 1. Introduction

Epoxy resin is a thermosetting resin that can form a highly cross-linked network by reacting with compounds containing amine, carboxylic acid, anhydride, or phenol or alcohol groups [1,2,3,4,5]. It is widely used as an insulating film material in the integrated circuit and chip industry [6,7,8]. With the rapid development of downstream substrate materials such as semiconductor packaging substrates, high-frequency circuit substrates, and micro-circuit substrates, the integration and precision of integrated circuits continue to improve, and a series of problems such as capacitor delay, crosstalk interference, increased energy consumption, poor thermal conductivity, signal transmission loss, and delay become more prominent [9,10,11]. The performance of ordinary epoxy resin is no longer sufficient to meet the requirements. Therefore, researchers are dedicated to developing a new type of epoxy resin system with a low dielectric constant and excellent overall performance. Current research is focused on two aspects: molecular design of the epoxy resin matrix and reinforcement with fillers [12,13].

The core idea of modifying the epoxy resin matrix is to introduce low-polarity atoms and structures. Li et al. [14] synthesized a novel fluorinated epoxy resin (diglycidol ether of 2,4-bis(1,1,1,3,3,3-hexafluoroisopropyl)fluorobenzene (FB–EP)) with 45.7 wt.% fluorine content was synthesized from fluorobenzene, epichlorohydrin, and hexafluoroacetone. De, B. et al. [15] prepared hyperbranched epoxy resins by condensation polymerization of pentaerythritol and diglycidyl ether of bisphenol-A, which exhibited good dielectric and mechanical properties. Wang et al. [16] designed and synthesized a new type of hyperbranched polymer that was designed and prepared with flexible chain blocking. The study showed that the resulting epoxy resin system had a minimum dielectric constant of 2.73 (10 MHz), a minimum dielectric loss of 0.002 (10 MHz), and an impact strength of 29.8 kJ/mm^2^. While the introduction of fluorine atoms could effectively improve the dielectric properties of the system, it may lead to environmental issues. On the other hand, hyperbranched molecules could improve the mechanical properties of the system, but their low crosslinking density after curing could lower the glass transition temperature of the system.

Dicyclopentadiene (DCPD) is a low-polarity structure with a large carbon skeleton, which gives it characteristics of low polarity and low hygroscopicity [17,18]. Hwang et al. [19] synthesized a DCPD–PPO phenolic resin with end hydroxyl groups and low molecular weight, which was used for the modification of an epoxy/4,4′-diaminodiphenylmethane (DDM) network system. The experimental results indicated that the dielectric constant of the DCPD–PPO epoxy resin system with a 40 wt.% content was 2.63, and the dielectric loss was 0.0104. Zou et al. [20] employed an anhydride-cured epoxy resin containing a DCPD structure. The experimental results demonstrated that the cured epoxy resin system had a glass transition temperature of 273.9 °C and a coefficient of thermal expansion of 13 × 10^−6^/°C. Li et al. [21] prepared four dicyclopentadiene-derived polyarylates (26–P, 26–M, 236–P, and 236–M) from 2,6-dimethyl (or 2,3,6-trimethyl) phenol-dicyclopentadiene adduct with terephthaloyl (or isophthaloyl) chloride by high-temperature solution polymerization. There was still room for improvement in the dielectric and thermal properties of the aforementioned DCPD epoxy resin system.

In addition to introducing low-polarity groups, reducing high-polarity groups is also a direction for the modification of epoxy resin systems. Hydroxyl groups are highly polar functional groups in epoxy resin systems. Activated ester is a type of curing agent that does not produce hydroxyl groups after reacting with epoxy resin [22,23]. It can reduce the content of hydroxyl groups in the resin and effectively improve the dielectric properties of the epoxy resin system. Meng et al. [24] prepared benzene-1,3,5-triyl tribenzoate (TBB), both 3,5-bis(benzoyloxy)benzoate-terminated poly (aryl ether ketone) oligomers (BPAPK and TMPK), containing active ester (Ph–O–(C=O)– structure), which were used as curing agents for dicyclopentadiene novoalc epoxy (DCPD). The experimental results showed that the glass transition temperature of the cured TMPK/DCPD was 218 °C, the dielectric constant was 2.75 (1 MHz), and the dielectric loss was 0.0085 (1 MHz). Yu et al. [25] synthesized dimer acid diglycidyl ester (DAGE) using a two-step method from dimer acid and used it to cure bisphenol A epoxy resin. The experimental results showed that the tensile strength and fracture elongation increased by 17.2% and 13.4%, respectively, when the DAGE content was 5 wt.%. Chen et al. [26] investigated the reaction mechanism between methyl methacrylate and bisphenol A epoxy resin and tested the dielectric constant of the resin system to be 2.924 at 1 GHz. The findings of these investigations indicated that active esters possessed the capability to undergo crosslinking reactions with epoxy resins, thereby resulting in the attainment of desirable dielectric properties.

The above research shows that ester curing agents can undergo crosslinking reactions with epoxy resins, ultimately leading to good dielectric properties. Ethyl phenylacetate is an ester curing agent that is commonly used in self-healing materials [27,28], but there is no research on its application in improving dielectric properties.

Various reinforcing materials such as polyhedral oligomeric silsesquioxane (POSS) [29], glass fibers/carbon nanotubes [30], graphene [31], TiO_2_ [32], silica [33], and hexagonal boron nitride [34] have significantly improved the mechanical, thermal, and dielectric properties of epoxy resin systems. However, filler aggregation remains a challenging issue that affects the performance of the resin system.

Gao et al. [35] incorporated HGM into the epoxy resin system to produce HGM/epoxy composite materials. The experimental results showed that the dielectric constant of the composite acoustic material was close to 1 and the dielectric loss was around 0.025 when the HGM content was 20 wt.%, which were 87% and 45% lower than those of the base epoxy resin, respectively. M. Imran et al. [36,37] utilized HGM to fill epoxy resins and achieved satisfactory mechanical performance. Zhang et al. [38] prepared low dielectric constant composite materials containing epoxy resin and HGM through the encapsulation of KH550 and grafting POSS. The experimental results demonstrated that the composite material exhibited an extremely low dielectric constant of 2.59 and a dielectric loss of 0.0145 when the filler content was at 20 weight percent. Furthermore, HGM displayed excellent compatibility and dielectric properties within the resin system.

Many studies have pointed out the excellent dispersibility and dielectric properties of HGM in epoxy resin systems [39,40,41] However, there is no report on the use of HGM to modify epoxy resins cured with ethyl phenylacetate.

The present study utilizes an ethyl phenylacetate-cured dicyclopentadiene-type epoxy resin and incorporates KH550-treated hollow silica particles to fabricate a composite material displaying outstanding comprehensive performance. Furthermore, the investigation delves into the mechanism by which the HGM affects the properties of the composite material.

## 2. Materials and Methods

### 2.1. Materials

Commercial hollow glass microsphere (HGM, 15 μm), a commercial DCPD epoxy resin (epoxy equivalent = 253–268 g/eq), commercial ethyl phenylacetate, commercial 4-dimethylaminopyridine (DMAP), and commercial γ-Aminopropyl triethoxysilane (KH550).

### 2.2. Preprocessing of HGM

For the experiment, KH550 silane coupling agent was selected to treat the surface of HGM. A KH550 ethanol dilute solution was prepared by mixing anhydrous ethanol, water, and KH550 in a ratio of 98:1:1, which was stirred until homogeneous and was then set aside for later use.

The required amount of HGM for the experiment was weighed and washed with an appropriate amount of ethanol to remove surface organic impurities. The washed HGM was placed in an 80 °C oven for 6 h to obtain dry HGM. The dry HGM was then added to the prepared KH550 ethanol solution and stirred evenly for 1 h with ultrasonic treatment followed by 1 h of standing time. After standing, the treated HGM was placed in an 80 °C oven for 6 h to obtain dry treated HGM.

### 2.3. Preparation of DSC Samples of Uncured Ethyl Phenylacetate/DCPD Epoxy Resin

The amount of ethyl phenylacetate and DCPD epoxy resin was taken in a 1:1 ratio based on the epoxy equivalent and ester group. First, we melted the DCPD epoxy resin in a beaker at 75 °C, then added the appropriate amount of ethyl phenylacetate, and stirred for 10 min. Next, we added 1 wt.% of the 4-dimethylaminopyridine and stirred slowly for 5 min. After stirring evenly, we let it stand and cool down.

### 2.4. Preparation of HGM/DCPD Epoxy Resin Composite Material

**Figure 1:**
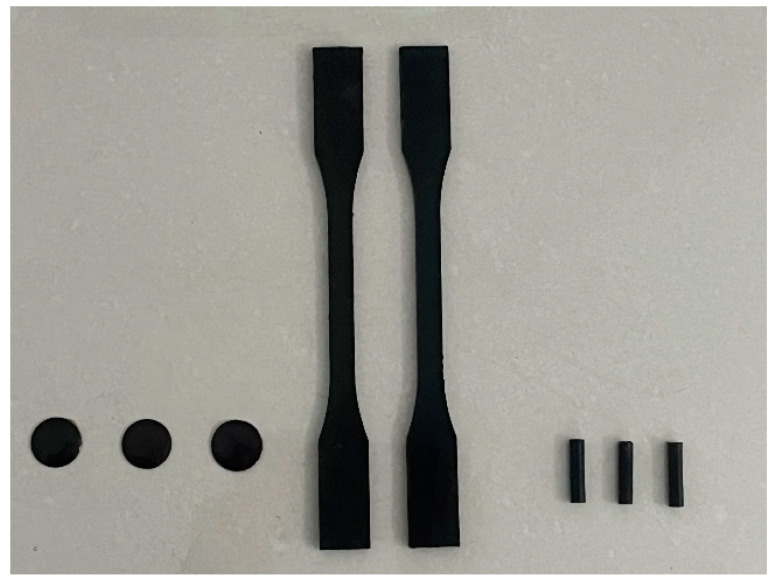
The composite materials were fabricated into dielectric samples, tensile samples, and thermal expansion samples using molds.

In this experiment, composite materials were prepared with different HGM addition amounts of 3, 5, 7, and 10 wt.%. As an example, the preparation process for the composite material with a 3 wt.% HGM content will be described. The quantities of ethyl phenylacetate and DCPD epoxy resin were calculated based on the equivalent amount of epoxy value and taken out for later use in a 1:1 equivalent ratio. After weighing the DCPD epoxy resin, it was poured into a beaker and heated to 75 °C to melt. Then, the corresponding amount of ethyl phenylacetate curing agent was added and stirred for 10 min until it was homogeneous. Next, 3 wt.% of pre-treated HGM was added and stirred for another 10 min. In total, 1 wt.% of 4-dimethylaminopyridine was added slowly and stirred for 5 min. The mixture was poured into a preheated mold. The mixture was cured at 82.6 °C for 2 h, then at 117.3 °C for 2 h, and, finally, at 135 °C for 2 h. The mixture was slowly cooled to room temperature with the furnace and then removed from the mold. The produced composite material is shown in Figure 1.

### 2.5. Characterization

Weigh about 10mg of the prepared DSC sample. Using a TA Q2000 differential scanning calorimeter in a nitrogen atmosphere, set the heating rate to 5 °C/min, 10 °C/min, 15 °C/min, and 20 °C/min, respectively. The scanning temperature range was from 25–260 °C. After scanning, process the obtained differential scanning curve, and record the initial reaction temperature (T_i_), peak temperature (T_p_), and final reaction temperature (T_f_). Use Origin software to analyze the obtained curve and derive the characteristic reaction temperature. The coupling agent and hollow glass microspheres (HGM) reaction, alongside the curing process between ethyl phenylacetate and epoxy resin, was examined via Fourier transform infrared (FTIR) spectroscopy. The spectroscopic analyses were performed using a Thermo Nicolet 6700 spectrometer within the range of 4000 to 400 cm^−1^. The dielectric properties of the composite materials were evaluated via an impedance analyzer (4294A, Agilent Technologies Inc., Santa Clara, CA, USA). The samples were prepared as thin disks with a diameter of 6mm and a thickness of 0.5mm, with electrodes plated on both upper and lower surfaces. The frequency range of the measurements was set from 1 MHz to 10 MHz. The dielectric constant and dielectric loss of the samples were calculated using the principle of parallel plate capacitor at room temperature. The thermal expansion coefficient and glass transition temperature of the composite material were measured using a thermal expansion instrument (Dil 402, NETZSCH, Free State of Bavaria, GER). The samples were prepared into standard samples with a diameter of 6 mm and a height of 25 mm. The measurement was carried out in a nitrogen atmosphere, with a heating rate of 10 ℃/min, and the temperature range was from 25 °C to 260 °C. The thermal conductivity of the composite material was measured using the Hot Disk TPS2500S thermal conductivity meter. The sample was prepared as a 3 mm thick, 20 mm × 20 mm plate, and the thermal conductivity was measured using the transient plane source (TPS) method in air. Samples were prepared for tensile testing of the casting performance of the resin according to the GB/T 2568-1995 standard using a DDL100 electronic universal testing machine. The test was conducted with a speed of 2 mm/min, and at least three samples were prepared for each composite material with the same HGM content to calculate the average value. The tensile modulus was calculated from the stress–strain curve.

## 3. Results and Discussion

### 3.1. Study of the Curing Reaction Characteristic Temperature

**Figure 2:**
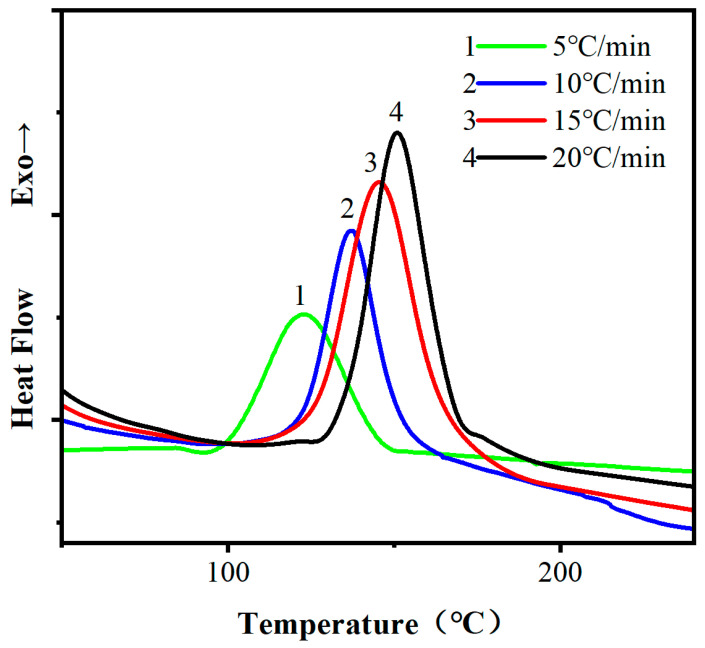
Non-isothermal DSC curve of ethyl phenylacetate cured DCPD epoxy resin.

Figure 2 shows the curing reaction curves of the mixture of ethyl phenylacetate and DCPD epoxy resin at different heating rates. As shown in Figure 2, a significant exothermic peak was observed in the DSC curves of the ethyl phenylacetate/DCPD epoxy resin system at different heating rates. With the increase in heating rate, the onset temperature of exothermic reaction gradually increased, and the peak of the exothermic reaction shifted towards higher temperature and became sharper.

As the heating rate of the system increases, the amount of heat generated per unit time increases, the temperature change per unit time increases, the thermal effect per unit time increases, and the temperature difference increases. Therefore, the exothermic peak moves towards the high temperature direction. At the same time, an increase in heating rate equals an increase in curing temperature, and thus the reaction time is shortened. This explains the gradually sharper peak shape of the exothermic peak. Additionally, the curing reaction rate between DCPD epoxy resin and ethyl phenylacetate also increases, resulting in a shorter reaction time.

**Figure 3:**
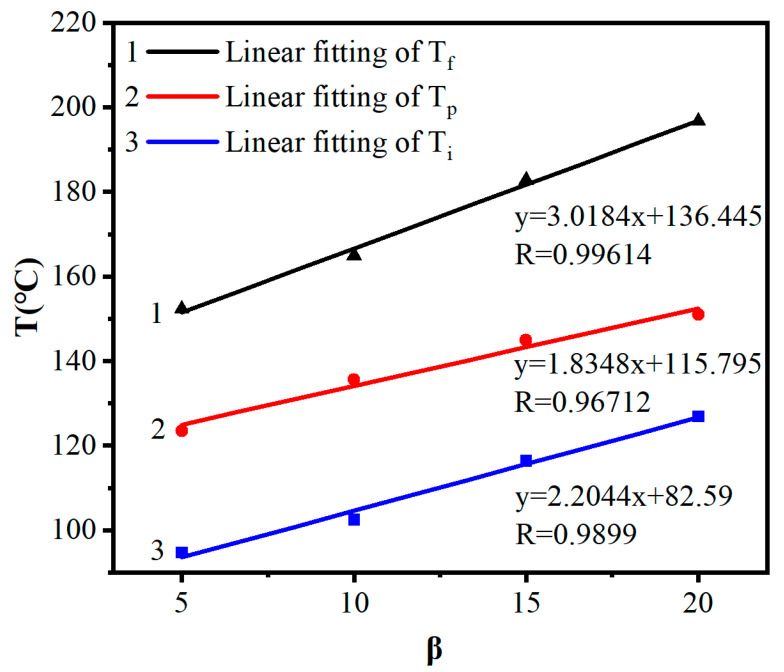
Linear relationship between T_i_, T_p_, T_f_, and β.

Setting T_i_ as the starting temperature of the exothermic peak, T_p_ as the peak temperature, and T_f_ as the endpoint temperature, they correspond to the gelation temperature, curing temperature, and post-curing temperature in the epoxy resin curing process. Based on the DSC data in Figure 2, a characteristic temperature table for the curing of ethyl phenylacetate and DCPD epoxy resin was made, as shown in Table 1. By plotting T_i_, T_p_, and T_f_ against the heating rate β according to Table 1, a linear regression was performed on the data, as shown in Figure 3. When β was extrapolated to 0, the gelation temperature of the system was found to be 82.6 °C, the curing temperature was 117.3 °C, and the post-curing temperature was 135.2 °C.

### 3.2. Fourier Transform Infrared (FTIR) Spectroscopy Analysis

**Figure 4:**
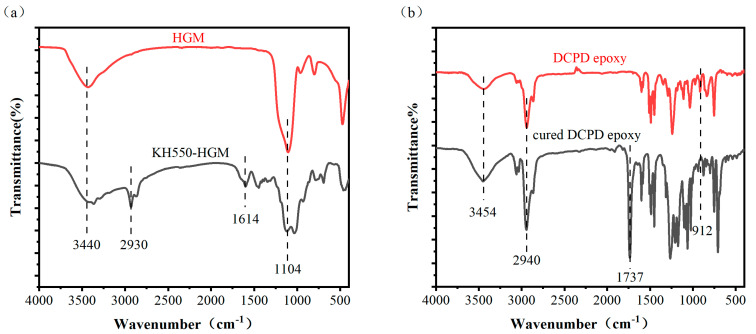
Shows the Fourier transform infrared spectra of (**a**) HGM and KH550-HGM, and (**b**) DCPD epoxy resin and cured DCPD epoxy resin with ethyl phenylacetate.

Figure 4 shows the Fourier transform infrared (FTIR) spectra of the HGM before and after pretreatment and the DCPD epoxy resin before and after curing. As shown in Figure 4a, for the HGM, there is a characteristic absorption peak of Si–OH at 3440 cm^−1^ and a peak of Si–O–Si at 1104 cm^−1^. The infrared spectrum of KH550-HGM exhibits characteristic absorption peaks at 2930 cm^−1^ and 1614 cm^−1^, corresponding to the C–H and N–H bonds, respectively. During the pre-treatment process, the ethoxy groups on KH550 are first hydrolyzed into hydroxyl groups in a mixed solution of ethanol and water and then combined with HGM. The presence of C–H and N–H bonds in the infrared spectrum of dried HGM indicates the existence of KH550’s amino propyl group on the surface of HGM particles, demonstrating the successful connection between KH550 and HGM during the pre-treatment process. This confirms that the pre-treatment of HGM was effective.

As shown in Figure 4b, for the DCPD epoxy resin, there is a hydroxyl absorption peak at 3454 cm^−1^, a C–H stretching peak at 2940 cm^−1^, and an epoxy group absorption peak at 912 cm^−1^. After curing, a carbonyl absorption peak of the ester group appears at 1737 cm^−1^, and multiple C–O–C absorption peaks are observed in the 1000–1300 cm^−1^ range. The epoxy group absorption peak at 912 cm^−1^ disappears completely. During the curing reaction between the epoxy resin and the phenylethyl acetate, the epoxy groups of the DCPD epoxy resin react with the ester groups of the phenylethyl acetate to generate new ester groups. The curing process is completed when all the epoxy groups have reacted, and the epoxy resins are interconnected with each other to form a three-dimensional network structure.

Based on the above reaction process, the characteristic absorption peak of the epoxy group of DCPD epoxy resin at 912 cm^−1^ in Figure 4b disappears in the cured DCPD epoxy resin, and a new absorption peak at 1737 cm^−1^ appears due to the carbonyl group in the ester, indicating that the DCPD epoxy resin has undergone a complete curing reaction with ethyl phenylacetate. The broadening of the absorption peak at 3454 cm^−1^ for hydroxyl groups may be attributed to the introduction of a small amount of hydroxyl groups in the system due to the addition of 4-dimethylaminopyridine and KH550.

### 3.3. Thermal Conductivity Analysis

**Figure 5:**
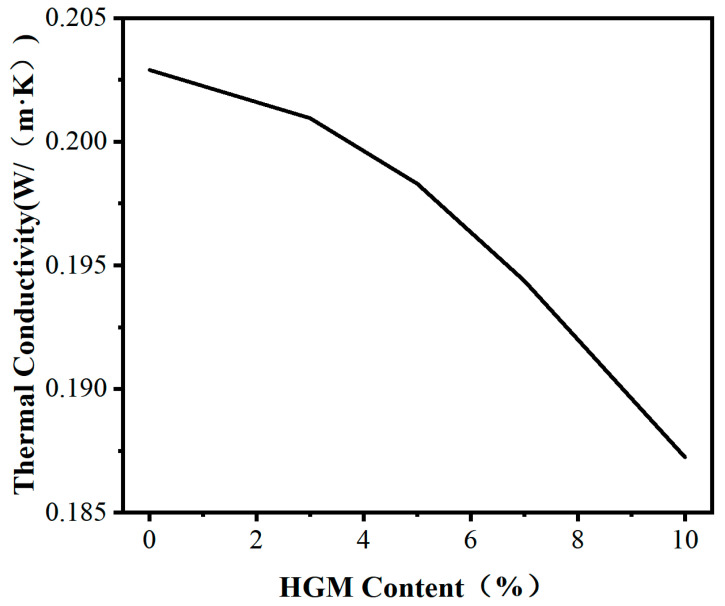
Influence of different HGM contents on the thermal conductivity of cured epoxy resin.

Figure 5 illustrates the impact of different HGM contents on the thermal conductivity of HGM/DCPD epoxy resin composites. As depicted, with the increase in HGM content, the thermal conductivity of the composite material shows a decreasing trend. Without the addition of HGM, the thermal conductivity of the composite material matrix is 0.2029 Wm^−1^ k^−1^. When the HGM content is 10 wt.%, the thermal conductivity is the lowest at 0.1872 Wm^−1^ k^−1^, representing a decrease of 7.73% compared to the matrix. The primary reason for this phenomenon is the increase in HGM content, which not only lowers its thermal conductivity but also results in the fracture of the thermal conduction path in the composite material.

In the epoxy resin system used in this experiment, there are three components, resin, HGM, and air, each with different thermal conductivities. Air has the poorest thermal conductivity and is wrapped by the outer layer of silica, making it difficult to form a thermal path. Compared to the resin, silica has stronger thermal conductivity, but, due to insufficient addition, the distance between particles is far apart, and they are blocked by the resin from forming a complete thermal path, which cannot improve the thermal conductivity. Therefore, the thermal function of the composite material is mainly carried by the base resin. On this basis, HGM containing air is dispersed in the system. When the heat flow passes through the resin, it encounters HGM particles, and the thin silica wall and internal air cannot help with thermal conduction. The heat flow can only bypass the HGM particles through the side resin group, which prolongs the heat transfer path in the material and further deteriorates the thermal conductivity of the composite material. The above analysis is consistent with the result shown in Figure 5, where, with the increase in HGM content, the rate of decrease in thermal conductivity becomes faster.

### 3.4. Analysis of Dielectric Properties

**Figure 6:**
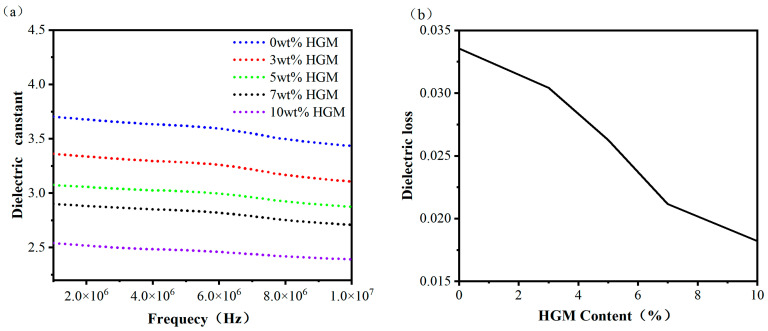
(**a**) Influence of different HGM contents on the dielectric constant of cured epoxy resin within the frequency range of 1–10 MHz. (**b**) Influence of different HGM contents on the dielectric loss of cured epoxy resin.

Figure 6 illustrates the effects of different HGM contents on the dielectric constant and dielectric loss of cured epoxy resin at different frequencies. As shown in Figure 6a, the addition of HGM to the epoxy resin composite results in a decrease in the dielectric constant. The dielectric constant of the composite material without HGM is 3.43 at a frequency of 10 MHz and 3.7 at 1 MHz. However, with 10 wt.% HGM content, the dielectric constant of the composite material is reduced by 30.3% and 31.3% at frequencies of 10 MHz and 1 MHz, respectively, with values of 2.39 and 2.54. In fact, the cured epoxy resin prepared by conventional methods typically exhibits a dielectric constant exceeding 4.0 [42,43]. The dielectric constant of the cured epoxy resin without HGM used in this experiment has already reached 3.7. The reason for the decrease in the dielectric constant of the HGM/DCPD epoxy resin composite material with the increase in HGM content is due to the bicyclic structure of the DCPD epoxy resin. This large skeleton structure consisting of 10 carbon atoms can increase the molar volume and reduce the polarity of the material, resulting in a decrease in the dielectric constant. Additionally, the ester-based curing agent selected for this experiment reacts with the epoxy resin to generate ester groups, which have lower polarities than the hydroxyl groups generated by common curing agents, further reducing the dielectric constant of the material. Therefore, the dielectric performance of the matrix resin is better than that of the general epoxy resin system. The addition of HGM significantly lowers the dielectric constant of the composite material mainly because HGM contains air, and dry air is an excellent dielectric with a dielectric constant typically around one. This low-dielectric-constant additive dispersed in the composite material plays a significant role in reducing the dielectric constant of the material.

As shown in Figure 6b, at 10 MHz, the addition of HGM to the epoxy resin leads to a decrease in the dielectric loss of the HGM/DCPD epoxy resin composite. The dielectric loss of the matrix resin is 0.034, while the lowest dielectric loss of the composite is achieved when the HGM content is 10 wt.%, which is 0.018, representing a reduction of 47% compared to the matrix. The factors affecting the dielectric loss of the composite material are almost the same as those affecting the dielectric constant. This is also due to the low dielectric properties of HGM particles, which reduces the strength of the internal electric field generated by the external electric field in the system, thus reducing the dielectric loss of the material.

### 3.5. Analysis of Thermal Expansion Coefficient

**Figure 7:**
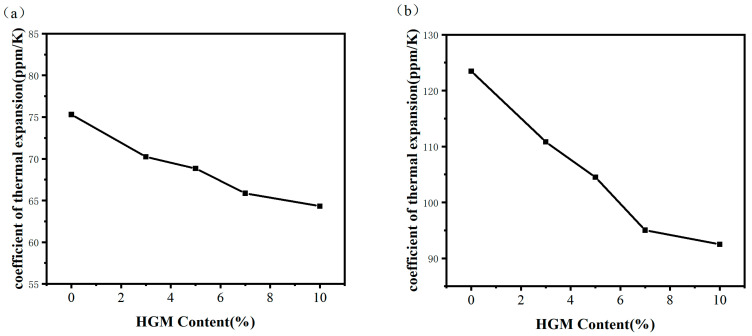
The impact of different HGM contents on the coefficient of thermal expansion of cured epoxy resin at (**a**) 50–150 °C temperature range and (**b**) 150–250 °C temperature range.

Resin films used as packaging materials require lower coefficients of thermal expansion to prevent phenomena such as curling and cracking during service [34]. Figure 7 illustrates the effect of different HGM contents on the thermal expansion coefficient of cured epoxy resin at temperatures between 50–150 °C and 150–250 °C. As shown in Figure 7a, within the temperature range of 50–150 °C, the thermal expansion coefficient of the HGM/DCPD epoxy resin composite material decreases with increasing HGM content. When no HGM is added, the thermal expansion coefficient of the base resin is 75.31 ppm/K. When the HGM content is 10 wt.%, the thermal expansion coefficient of the composite material is 64.31 ppm/K, which is 25.1% lower than that of the base material. As shown in Figure 7b, within the temperature range of 150–250 °C, the thermal expansion coefficient of the HGM/DCPD epoxy resin composite material also decreases with increasing HGM content. When no HGM is added, the thermal expansion coefficient of the base resin is 123.47 ppm/K. When the HGM content is 10 wt.%, the thermal expansion coefficient of the composite material is 92.49 ppm/K, which is 25.1% lower than that of the base material. From Figure 7, it can be observed that the thermal expansion coefficient of the composite material is generally lower below the glass transition temperature, and the decrease in thermal expansion coefficient is significant with the increase in HGM content from 0 to 10 wt.% above the glass transition temperature.

The main reason for the decrease in the average thermal expansion coefficient of the HGM/DCPD epoxy resin composite material system with increasing HGM content is due to the low thermal expansion coefficient of HGM itself. On the other hand, below the glass transition temperature, the molecular chains of the HGM/DCPD epoxy resin composite material cannot move, and the thermal expansion is mainly caused by the thermal vibration of the atomic groups, resulting in a low thermal expansion coefficient. Furthermore, at high temperatures, the molecular motion of the composite material becomes more intense, exceeding the glass transition temperature, and the polymer chains gain motion ability, leading to an increase in the expansion coefficient of the composite material. Additionally, HGM particles dispersed in the resin matrix occupy space and limit the movement ability of polymer chains, thereby reducing the thermal expansion coefficient. Below the glass transition temperature, the average thermal expansion coefficient of the composite material system is mainly affected by the material composition itself. Above the glass transition temperature, the average thermal expansion coefficient of the composite material system is also influenced by the motion of polymer chains. HGM particles can effectively reduce this effect, leading to a relatively large reduction in the average thermal expansion coefficient.

**Figure 8:**
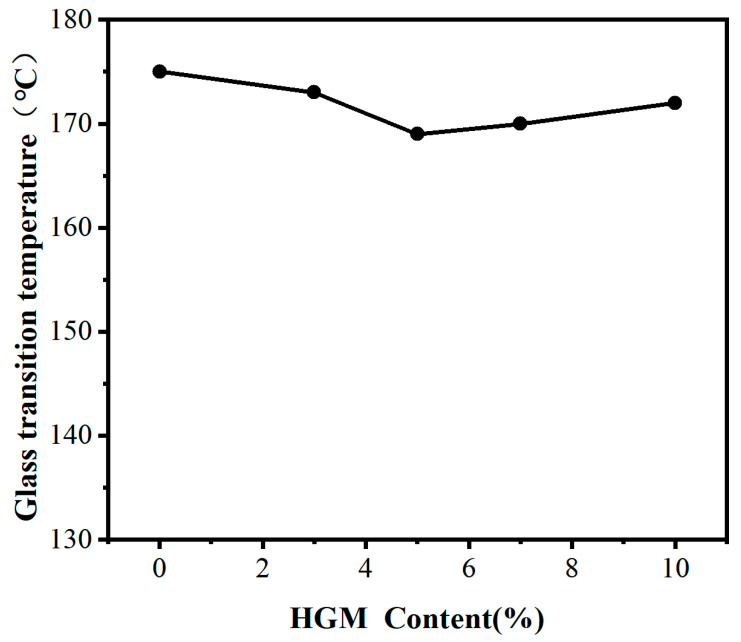
The effect of different HGM contents on the glass transition temperature of cured epoxy resin.

Figure 8 illustrates the effect of different HGM contents on the glass transition temperature of cured epoxy resin. As shown in Figure 8, it can be observed that the glass transition temperature of the HGM/bicyclic system composite material mainly fluctuates between 169 °C and 175 °C. The glass transition temperature of the base resin without HGM is 175 °C. When the HGM content is 10 wt.%, the glass transition temperature of the composite material is 172 °C. With the increase in HGM content, the glass transition temperature of the material shows a trend in first decreasing and then increasing. When the HGM content is 5 wt.%, the glass transition temperature is 169 °C, which is 3.4% lower than the highest point of 175 °C.

The addition of HGM particles affects the flowability of the polymer chains in the resin system. The deformed polymer chains make some affected segments more mobile, leading to a decrease in the glass transition temperature. As the HGM content increases, a large number of HGM particles occupy the polymer chain space, limiting the movement of the polymer chain segments, resulting in an increase in the glass transition temperature of the composite material system. This leads to a phenomenon of initially decreasing and then increasing glass transition temperature.

### 3.6. Analysis of Glass Transition Temperature

**Figure 9:**
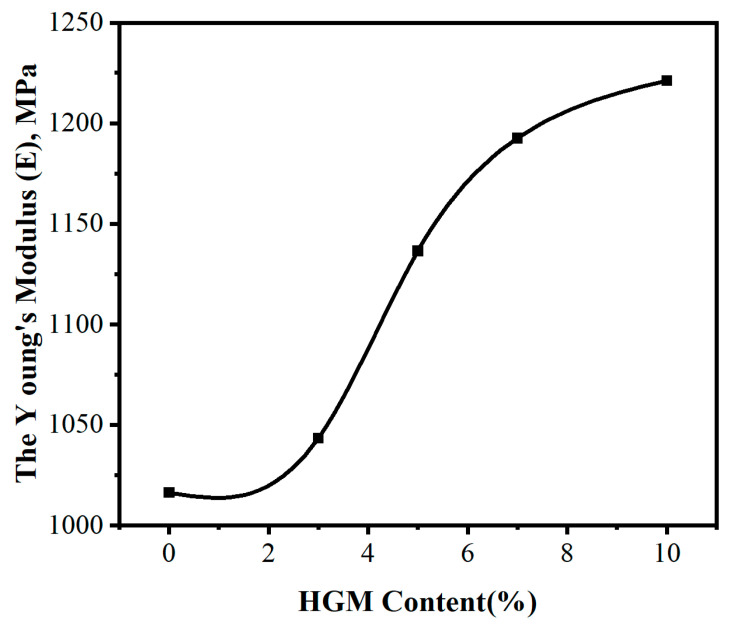
The effect of different HGM content on the elastic modulus of cured epoxy resin.

Figure 9 illustrates the influence of different HGM contents on the elastic modulus of cured HGM/DCPD epoxy resin composites. As shown in Figure 9, the elastic modulus of the composite material shows an increasing trend with the increase in HGM content. When no HGM is added, the elastic modulus of the matrix resin is 1016.4 MPa. When the HGM content is 10 wt.%, the elastic modulus of the composite material is 1221.13 MPa, which is 20.14% higher than that of the matrix.

The addition of HGM affects the elastic modulus of cured epoxy resin in three ways. Firstly, the mobility of molecular chains is the primary determinant of the elastic modulus of epoxy resin, with greater mobility leading to lower elastic modulus. The addition of HGM impedes the mobility of molecular chains, thus increasing the elastic modulus of the epoxy resin. Secondly, the addition of HGM reduces the free volume of the system, which impedes the movement of molecular chains and thereby increases the elastic modulus of the composite material. Finally, HGM is coupled to the resin molecule by KH550, and the reaction of KH550 amino groups with epoxy groups increases the cross-linking density of the system, which also increases the elastic modulus of the composite material. However, as the HGM content increases, the rate of increase in the elastic modulus of the composite material slows down. This is mainly due to the increase in system viscosity caused by the increase in HGM content, resulting in the production of more defects such as air pockets and cracks after curing, which decrease the elastic modulus of the composite material and thus reduce the rate of increase.

## 4. Conclusions

This study employed ethyl phenylacetate to cure a DCPD epoxy resin and incorporated hollow glass microsphere treated with KH550 into the system, resulting in the fabrication of HGM/DCPD epoxy resin composites. The following conclusions were drawn:

(1) Ethyl phenylacetate can cure the DCPD epoxy resin.
(2) The suitable curing process is as follows: firstly, cure at 82.6 °C for 2 h, then raise the temperature to 117.3 °C, cure for 2 h, and, finally, cure at 135 °C for 2 h.
(3) The thermal conductivity of the composite material decreases with an increase in the content of HGM. At an HGM content of 10 wt.%, the thermal conductivity of the composite material is at its lowest, with a value of 0.1872 Wm^−1^ k^−1^, representing a 7.73% reduction compared to the matrix.
(4) The dielectric constant and dielectric loss of the composite material also decrease with the increase in HGM content. At a frequency of 10 MHz, the composite material has the lowest dielectric constant of 2.39, which is 30.3% lower than the matrix. The dielectric loss of the composite material is lowest at 10 wt.% HGM content, which is 0.018, representing a 47% reduction compared to the matrix.
(5) The coefficient of thermal expansion of the composite material decreases as the HGM content increases. In the temperature range of 50–150 °C, the composite material has the lowest coefficient of thermal expansion of 64.31 ppm/K when the HGM content is 10 wt.%. In the temperature range of 150–250 °C, the composite material has the lowest coefficient of thermal expansion of 92.49 ppm/K when the HGM content is 10 wt.%, which is 25.1% lower than that of the matrix.
(6) As the HGM content increases, the glass transition temperature of the composite material first decreases and then increases, with the highest point being 175 °C and the lowest point being 169 °C, with a maximum variation of 5%. This indicates that the glass transition temperature of the material is not greatly affected by the HGM content.
(7) The elastic modulus of the composite material increases with the increase in HGM content, and the growth rate is fast at first and then slows down. The highest value is 1221.13 MPa, which is 20.14% higher than that of the matrix.

This study demonstrates a new design approach for epoxy-resin-based composite materials, making a contribution to the development of low dielectric epoxy resin films.

The above conclusion indicates that the composite material prepared in this study has achieved good results in terms of dielectric and heat resistance. However, there are several issues that need to be addressed in future research. First, the cooling cycle during the curing reaction process should be explored. Secondly, considering the actual application scenarios of the material, the changes in electrical conductivity of the composite material at different frequencies need to be investigated. Finally, it is necessary to study the dielectric properties of the material at lower frequencies, analyze various polarization mechanisms through dielectric dispersion, and explore the corresponding relationship between the microstructure and dielectric properties of the composite material. These efforts will lay a foundation for future work to further enhance the performance of the material.

## Figures and Tables

**Table 1:** Characteristic temperatures of the ethyl phenylacetate/DCPD epoxy resin system at different heating rates.

Heating Rate β (°C/min)	T_i_ (°C)	T_p_ (°C)	T_f_ (°C)
5	94.76	123.45	152.33
10	102.56	135.63	164.45
15	116.36	144.9	182.7
20	126.68	150.94	196.62

## Data Availability

Data will be available upon request.
